# Cystoscopic removal of an intravesical gossypiboma mimicking a bladder mass: a case report

**DOI:** 10.1186/1752-1947-5-579

**Published:** 2011-12-16

**Authors:** Romeo Kansakar, Bhairab Kumar Hamal

**Affiliations:** 1 Gastrointestinal and Laparoscopic Surgery Unit, Department of Surgery, Bir Hospital, National Academy Of Medical Sciences, Mahaboudha, Kathmandu, Nepal; 2 Department of Surgery, Shree Birendra Hospital, Chauni, Kathmandu, Nepal

## Abstract

**Background:**

Intravesical retained surgical sponges are very rare and only a few cases have been removed by minimally invasive techniques.

**Case presentation:**

We report a case of an intravesical gossypiboma in a 71-year-old man from western Nepal, who presented with urinary retention and persistent lower urinary tract symptoms one year after open cystolithotomy. He was diagnosed with an intravesical mass using ultrasonography. The retained surgical sponge was found during cystoscopy and removed through endoscopy.

**Conclusion:**

Intravesical gossypibomas are rare and can mimic a bladder mass. This is one of the few reported cases of cystoscopic removal.

## Background

Gossypiboma denotes a foreign body made of cotton that is retained inside the patient during surgery [1]. Although it is an uncommon condition, it is the dread of every surgeon. It has been reported to occur following surgical procedures such as abdominal, thoracic, cardiovascular, orthopedic, urological and even neurosurgical operations [2-4]. It is estimated to occur in one in 100 to 3000 cases for all surgical interventions and one in 1000 to 1500 for intra-abdominal operations [5]. Preoperative diagnosis may be difficult due to nonspecific symptoms and inconclusive imaging findings [6]. There have been few reports of intravesical gossypibomas and even fewer reports of cystoscopic removal [7-9]. We report a case of a retained intravesical sponge following open cystolithotomy, with transurethral removal under cystoscopic guidance.

## Case presentation

**Figure 1:**
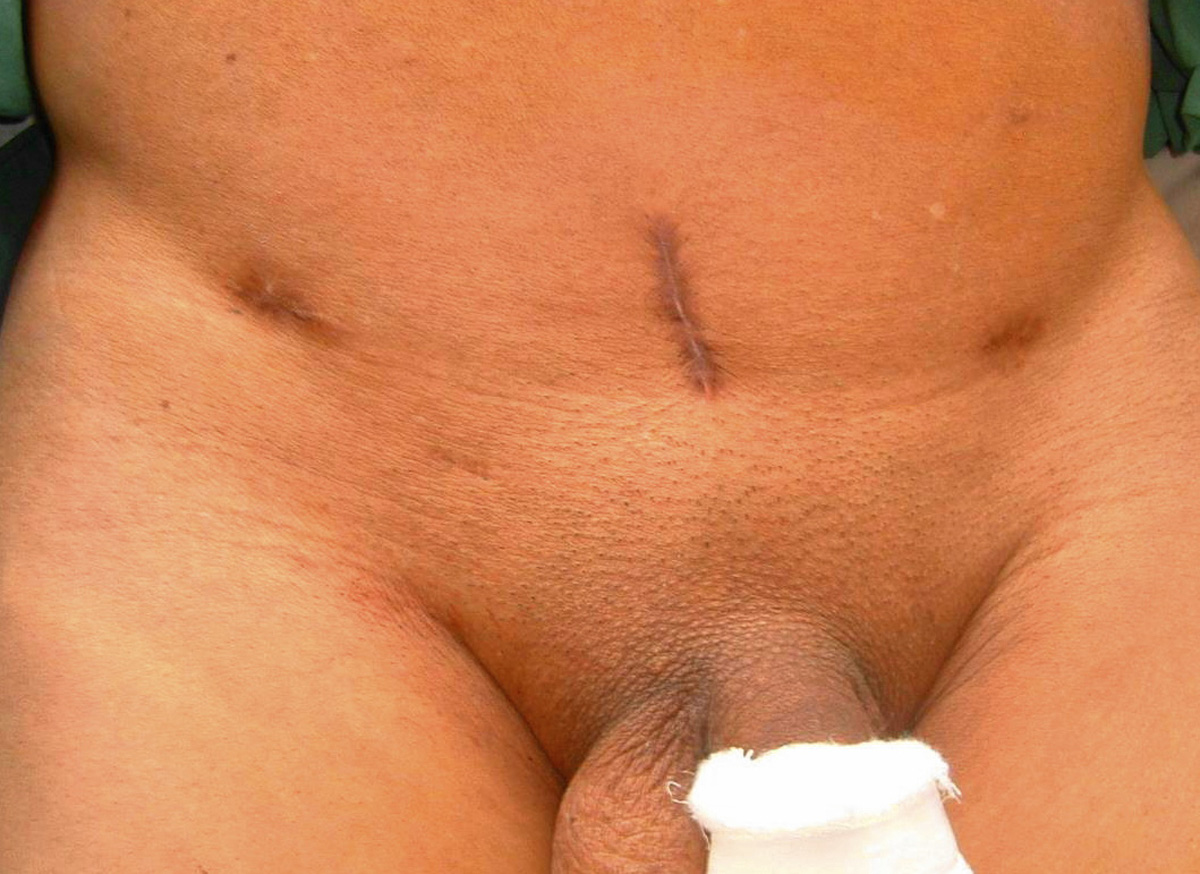
Preoperative open cystolithotomy scar

**Figure 2:**
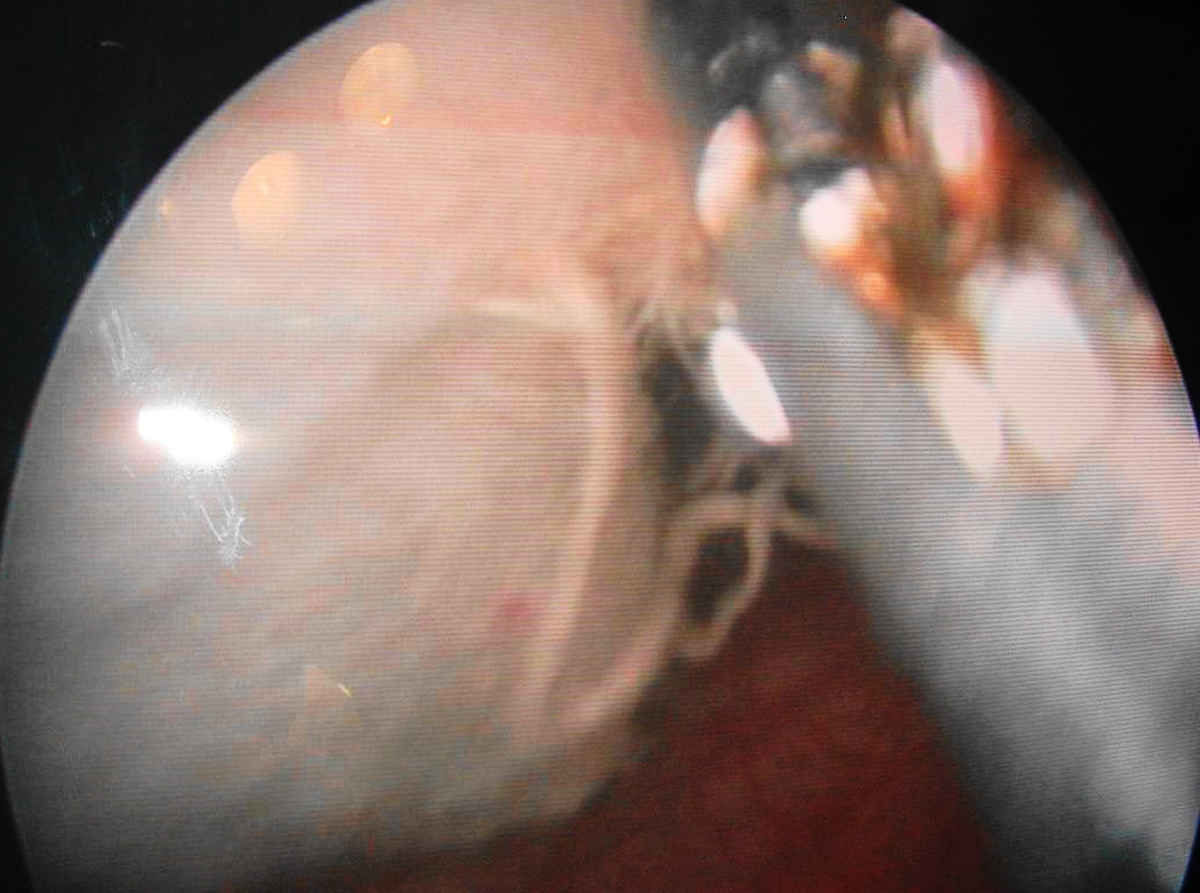
**Visualization of the surgical sponge after manipulation during cystoscopy**.

**Figure 3:**
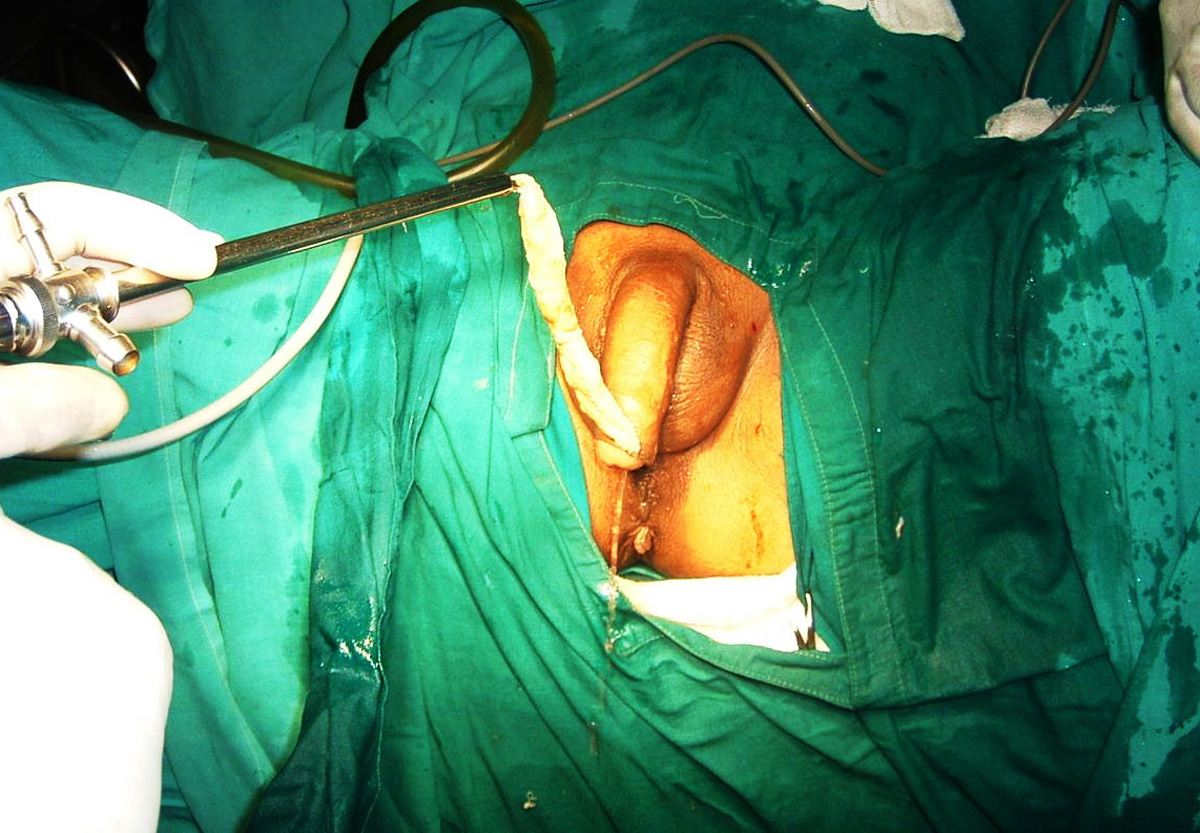
**Transurethral removal of the surgical sponge using a lithotrite**.

**Figure 4:**
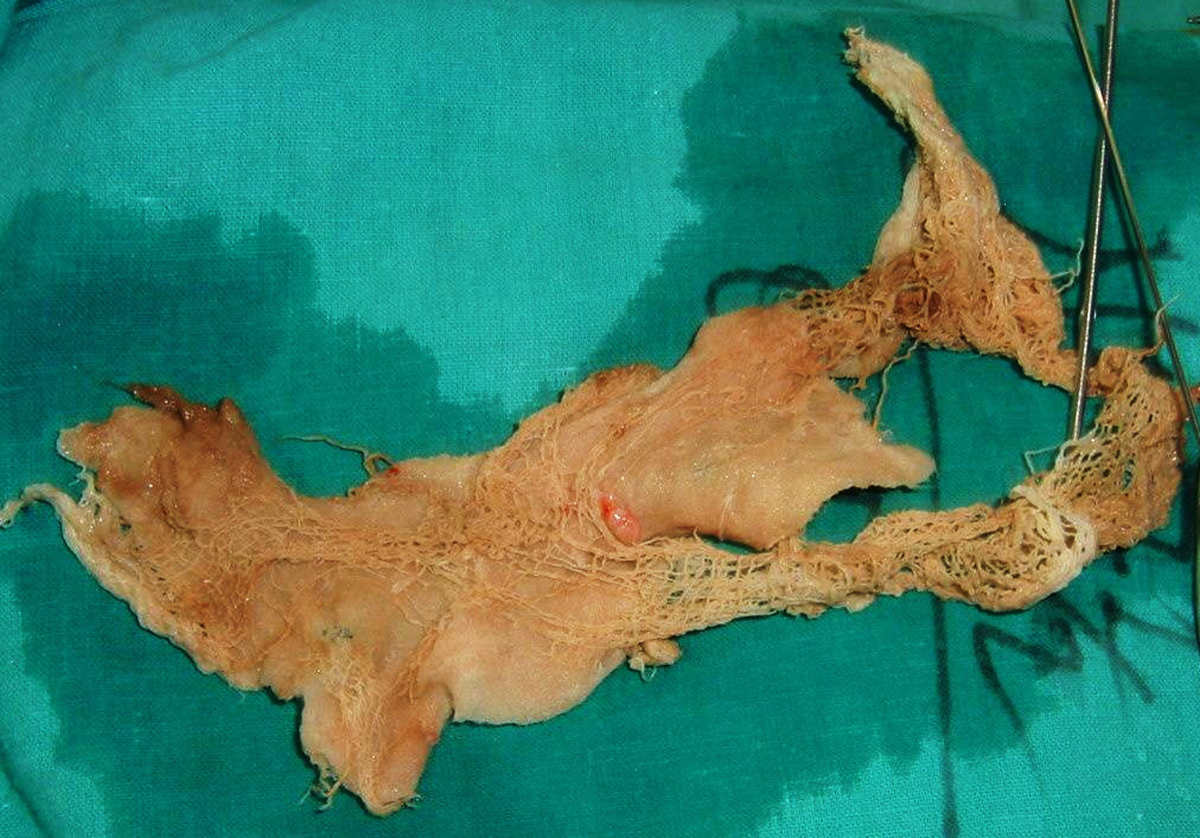
**The surgical sponge after removal**.

A 71-year-old man from western Nepal presented to us with a history of urinary retention for 20 days for which a urinary catheter was placed. He had been experiencing increased urinary frequency, dysuria, suprapubic pain, intermittent fever and hematuria for the last six months. He had undergone an open cystolithotomy for vesical calculus one year previously in a peripheral hospital. On examination, his vitals were stable with a lower midline scar (Figure 1) and mild tenderness over his lower suprapubic region, with a Foley catheter *in situ*. A urine analysis showed pyuria, with12 to 15 red blood cells, and a urine culture showed growth of *Proteus mirabilis *sensitive to nitrofurantoin. A renal function test and other investigations were within normal limits. An abdominal X-ray was unremarkable and ultrasound examination showed sludge in his urinary bladder, with a mass of 8 cm × 13 cm. Our patient was diagnosed with a bladder mass and cystoscopy was planned after a week of antibiotics. The cystoscopy revealed a large mass in the anterior wall of his bladder, mimicking a fungating bladder tumor. Upon further examination, pieces of cotton thread were evident and confirmed the mass to be a gossypiboma (Figure 2); there was no other bladder mass. Surgical gauze of around 6 cm × 10 cm was removed piecemeal using Double J stent removing forceps and a lithotrite (Figures 3 and 4). His postoperative recovery was unremarkable.

## Discussion

Gossypiboma is rarely seen in clinical practice. Intra-abdominal gossypiboma is the most common. Intravesical gossypiboma is infrequently reported in the literature [9], probably in part due to the legal implications, setting up a vicious cycle of non-anticipation and misdiagnosis. There have also been reports of calcified retained vesical gauze mimicking bladder stones [8] and even spontaneous transurethral protrusion [7].

The preoperative diagnosis of retained gauze may be difficult, especially if radio opaque markers are not placed like in our case. Ultrasonography, computed tomography and magnetic resonance imaging can aid in diagnosis but still may be confusing [10]. The presence of brightly echogenic wavy structures showing posterior acoustic shadowing that change in parallel with the direction of the ultrasound beam has been reported as a diagnostic feature of a gossypiboma [11]. Computed tomography findings may be indistinguishable from those for an intra-abdominal mass or abscess, since air bubbles, calcification and contrast enhancement of the rim may be seen in both conditions. A gossypiboma may be misdiagnosed as a tumor, which may lead to unnecessary invasive diagnostic procedures or extensive surgery.

Once a gossypiboma is identified, it should be removed. Surgery had been the mainstay in the removal of foreign bodies for many years. However, various techniques have been applied for the removal of retained gauze, such as percutaneous, endoscopic and laparoscopic extraction and open surgery, depending on clinical presentations and facilities available. Nosher *et al. *described six patients in whom percutaneous extraction was successful [12]. Rafique described three cases of cystoscopic removal [13].

Clearly, prevention of this condition is preferable; this can be achieved by a meticulous count of surgical materials in addition to thorough exploration of the surgical site at the conclusion of surgery. Although textiles impregnated with radio-opaque markers are widely used by surgeons in the developed world, it is not practiced in our part of the world and thus diagnosis is still difficult. Radio-opaque markers should be routinely used and if in doubt, intraoperative radiologic screening should be done. Gossypiboma is a surgical mishap which can be avoided if guidelines for operative theatre record keeping are carefully followed. The surgical team should not unquestionably accept correct count reports, but should develop the habit of performing a brief but thorough routine post procedure wound body cavity exploration before closure. The routine use of radio-opaque markers, not currently used in our part of the world, is a must. Despite all the technologic advances of the 21st century, human fallibility remains. The possibility of gossypiboma exists even in modern medicine. As litigation is becoming ever more common for this avoidable problem, prevention is highly desirable. Among the many alternative methods of treatment, endoscopic removal is possible in some cases.

## Conclusion

Intravesical gossypibomas are rare and can mimic a bladder mass. This is one of the few reported cases where removal was performed with the help of cystoscopic guidance.

## Consent

Written informed consent was obtained from the patient for publication of this manuscript and any accompanying images. A copy of the written consent is available for review by the Editor-in-Chief of this journal.

## Competing interests

The authors declare that they have no competing interests.

## Authors' contributions

RK is the principal author and assistant during the procedure. He collected the history and information from the patient and was the principal writer and performed the literature review. BKH was the principal surgeon. He reviewed and corrected the article. Both authors read and approved the final manuscript.
